# Therapeutic Strategies Aimed at Improving Neuroplasticity in Alzheimer Disease

**DOI:** 10.3390/pharmaceutics15082052

**Published:** 2023-07-31

**Authors:** María F. Colavitta, Francisco J. Barrantes

**Affiliations:** 1 Laboratory of Molecular Neurobiology, Biomedical Research Institute (BIOMED), Universidad Católica Argentina (UCA)—National Scientific and Technical Research Council (CONICET), Buenos Aires C1107AAZ, Argentina; 2 Centro de Investigaciones en Psicología y Psicopedagogía (CIPP-UCA), Facultad de Psicología, Av. Alicia Moreau de Justo, Buenos Aires C1107AAZ, Argentina; mariaflorenciacol@uca.edu.ar

**Keywords:** Alzheimer disease, cognition, neuroplasticity, neurotransmitters, long-term potentiation, cognitive impairment: animal models, dementias, neurodegenerative diseases, neuroinflammation, therapeutics

## Abstract

Alzheimer disease (AD) is the most prevalent form of dementia among elderly people. Owing to its varied and multicausal etiopathology, intervention strategies have been highly diverse. Despite ongoing advances in the field, efficient therapies to mitigate AD symptoms or delay their progression are still of limited scope. Neuroplasticity, in broad terms the ability of the brain to modify its structure in response to external stimulation or damage, has received growing attention as a possible therapeutic target, since the disruption of plastic mechanisms in the brain appear to correlate with various forms of cognitive impairment present in AD patients. Several pre-clinical and clinical studies have attempted to enhance neuroplasticity via different mechanisms, for example, regulating glucose or lipid metabolism, targeting the activity of neurotransmitter systems, or addressing neuroinflammation. In this review, we first describe several structural and functional aspects of neuroplasticity. We then focus on the current status of pharmacological approaches to AD stemming from clinical trials targeting neuroplastic mechanisms in AD patients. This is followed by an analysis of analogous pharmacological interventions in animal models, according to their mechanisms of action.

## 1. Introduction

One of the characteristics of aging is the change in cognitive performance in a continuum that spans a wide range of severities, from subtle changes along “normal” aging to the more profound decline associated with neurodegenerative diseases. Some older adults present no apparent changes in cognition, while others exhibit so-called mild cognitive impairment (MCI), a condition that can be described as a minor decline in cognition, greater than that normally expected at the individual’s age but not sufficient to interfere greatly with their normal daily activities. A third set of individuals presents clear signs of severe cognitive compromise. MCI can, but does not always, develop into a more profound disorder, limiting normal daily functioning, at which stage the patient may present other symptoms that categorize the status known as dementia [1].

AD is the most common form of cognitive disorder in the elderly and frequently develops into full dementia. The disease significantly affects one or more cognitive domains of the patient, memory being the most impaired brain function [2]. This is the typical manifestation in most cases of AD, though the clinical phenotype and etiology may differ in individual presentations. There are two main categories of AD: sporadic and familial. Whilst to date no causative genes have been conclusively connected with the sporadic or late onset form of the disease (LOAD), several mutations have been identified in specific genes associated with the development of familial or inherited AD: amyloid precursor protein, presenilin-1, presenilin-2, and apolipoprotein E. Familial AD frequently manifests at earlier ages and is therefore referred to as early onset AD (EOAD); its clinical manifestations and progression are typically more aggressive than those of sporadic AD [3,4].

The most widely used experimental models of AD in studies on therapeutic strategies are transgenic rodent models that carry the mutations characteristically found in LOAD. In contrast, clinical trials usually address the EOAD form rather than LOAD, as the latter represents less than 5% of all cases [5]. There are, however, clinical trials involving patients at preclinical and clinical stages of familial AD to test for instance monoclonal antibody therapy, such as that involving solanezumab [6,7].

Neurofibrillary tangles and amyloid deposits are the two hallmarks of AD. These two postmortem neuropathological findings are believed to be preceded by more subtle subcellular and biochemical processes involving the constituent molecules: (i) tau, the microtubule-associated protein involved in axonal transport under physiological conditions, is translocated to the somatodendritic space and undergoes hyperphosphorylation, misfolding, and aggregation in AD, leading to the formation of neurofibrillary tangles; (ii) amyloid β (Aβ), a protein resulting from the hydrolytic cleavage of the amyloid precursor protein (APP), normally helps protect against infections and injuries, repairs leaks to the blood-brain barrier, and mediates synaptic transmission and plasticity. In AD, Aβ forms soluble oligomers that have a synaptotoxic effect, later forming extracellular deposits of amyloid plaques, found to be abundant in the brain cortex of AD patients [8]. In addition to the hyperphosphorylated tau and amyloid burden observed in most AD patients, there are other mechanisms that may be partly responsible for the observed decline in cognition. Of particular importance are alterations in neurotrophic signaling, cell survival, neurogenesis, and synaptic function, all of which are subjacent neuroplastic phenomena that can be significantly reduced or lost in AD [9], as discussed in this review. It has been hypothesized that functional alterations in neuroplasticity rather than epiphenomenological neuronal degeneration and death—reflected in the postmortem neurofibrillary tangles and hyperphosphorylated tau protein deposits—could be responsible for some of the cognitive impairments in AD, as well as in other neurodegenerative diseases [10,11,12]. Despite the strong correlation between plastic alterations and cognitive decline in AD, no effective treatments towards slowing down neuronal degeneration, stopping neuronal death, or enhancing the activity of surviving neurons have been found to date. Growing evidence points to the evaluation of dysfunctional neuroplasticity in experimental and clinical scenarios, as this general set of manifestations is increasingly considered a strong clinical correlate of the disease [13,14]. Pharmacological therapeutic strategies aimed at enhancing neuronal plasticity in AD are discussed in this review, with particular focus on studies that measure mechanisms of plasticity in AD patients or animal models of AD following pharmacological interventions. Articles were searched using the PubMed database and clinicaltrials.gov, selecting only those reporting experimental measurements of neuroplasticity.

## 2. The Multiple Facets of Neuroplasticity

Neuroplasticity, also called brain plasticity or simply plasticity, refers to the combination of processes that generate adaptive changes in the brain following acquired experience or damage [15]. In adulthood, neuroplastic mechanisms tend to diminish. The ability to preserve plasticity is considered essential for healthy ageing, as it may constitute a protective factor against age-related conditions and even neurodegenerative diseases such as AD [16,17]. The reorganization of the brain that is inherent to neuroplasticity comprises various mechanisms, operationally classified as functional or structural [18], though this is in fact a misleading dichotomy since the two operate jointly. At the crossroad of behavioral and functional neuroplastic parameters we find mechanisms such as homologous area adaptation (a cognitive function is overtaken by a brain structure from the opposite hemisphere), cross-modal reassignment (brain areas accustomed to processing a specific kind of sensory input develop the ability to respond to an additional sensory input), compensatory masquerade (a cognitive function is allocated to a new area), and map expansion (a given functional brain region is expanded following repetitive stimulation) [19,20,21].

Structural plasticity usually refers to the morphological and developmental changes in neurons and synapses along neurodevelopment, i.e., the remodeling of neuronal circuits or synapses (e.g., synaptic pruning) or the generation of new nerve cells (i.e., neurogenesis) or synapses (i.e., synaptogenesis) either in developmental stages or in neuronal repair mechanisms [22,23]. Parameters such as number of new neurons, axon and dendritic length, number and stability of dendritic arborizations, and number and morphology of synaptic boutons, can be measured in post-mortem human or animal brain tissue [24]. The activity of neurons can be assessed at the level of networks in the resting state and under conditions of hypo- or hyper-connectivity. The latter can be studied indirectly in humans through nuclear magnetic resonance (NMR) imaging (MRI) or functional MRI (fMRI) [25].

**Figure 1:**
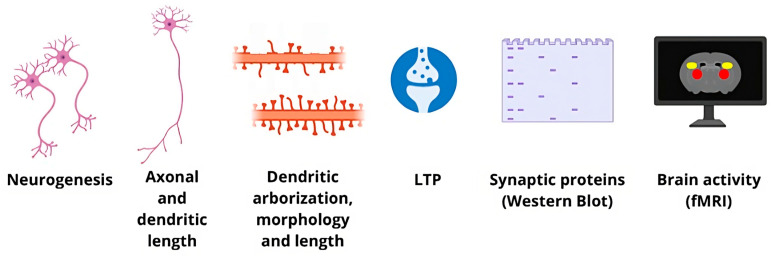
Schematic representation of different experimental measurements of neuroplasticity.

At the cellular level, electrophysiological measurements can provide information on the functional state of the individual neuron, e.g., whether it is in a status of long-term potentiation (LTP) or long-term depression (LTD), these measurements generally being obtained ex vivo or in vitro [26]. LTP refers to the long-lasting strengthening of connections between neurons after repetitive stimulation, a phenomenon that is strongly correlated with learning mechanisms and the consolidation of long-term memory [27]. It is generally accepted that continuous stimulation and strengthening through LTP can reach a ceiling effect, requiring a mechanism of synaptic weakening to be adopted: LTD enhances neuroplasticity by preventing synaptic connections from reaching this ceiling effect [28,29]. These measurements at the structural and functional level are summarized in Figure 1.

Measuring neuroplasticity in human subjects is obviously precluded by ethical and methodological considerations, calling for the use of proxy measures. Thus, measuring neuroplasticity was for many years restricted to the use of neuropsychological tests [30], such as the Battery of Learning Potential for Assessing Dementia [31] or the re-adapted Auditory Verbal Learning Test [32], and indirect biomarkers in serum and cerebrospinal fluid (CSF) [33,34,35,36]. CSF biomarkers of plasticity include: neurogranin, a postsynaptic protein involved in synaptic plasticity and LTP [37], whose levels are usually higher in AD patients [38]; synaptosome-associated protein-25 (SNAP-25), which participates in the control of synaptic plasticity [39] and is usually higher in AD patients [40]; brain-derived neurotrophic factor (BDNF), essential for memory formation and structural plasticity [41] and which is lower in MCI and AD patients [42,43]; and vascular endothelial growth factor (VEGF), a protein involved in the growth of blood vessels and delivery of glucose that has a role in enhancing neurogenesis and synaptic plasticity [33] (see Table 1). Functional MRI (fMRI) has revolutionized this field, making it possible to obtain information on the involvement of certain brain areas in, e.g., mnemonic, cognitive, and/or fear processing. The spatial- and time-resolution of this technique, however, still falls short of addressing the cell (neuron) or subcellular (synapse) levels, that is, measuring the neuronal and/or synaptic integrity of patients in vivo. None of the above measures lead to an unequivocal diagnostic of AD [44,45].

AD is characterized by two main pathological findings in post-mortem tissue: the deposition of amyloid-beta peptides (Aβ) and neurofibrillary tangles. Other features accompany these two necropsy findings, such as neuroinflammation, cell death, and synaptic loss [46]. The so-called amyloid hypothesis purported that Aβ deposition and consequent toxicity were the causative origins of the disease [47,48,49,50]. Despite its dominant influence, there is still no conclusive proof of the original hypothesis. Some authors suggest that AD could be primarily a disease of the synapse, whereby synaptic dysfunction leads to synaptic loss and, in consequence, to neurodegeneration [51]. Synaptic aberration occurs at early phases of AD, mainly in the mesotemporal regions of the brain [52]. These abnormalities may be caused by amyloid toxicity, though no agreement has been reached on whether synaptic alterations occur prior to the deposition of senile plaques or as a consequence of Aβ deposition [53,54]. Aβ is also thought to hamper LTP in the hippocampus [55] and to disrupt LTD function by preventing glutamate uptake [56]. The inhibition of LTP and enhancement of LTD leads to synaptic and dendritic shrinkage [57,58]. In the presence of AD pathology, however, especially during the early stages of the disease, the brain still possesses the ability to adapt and rewire itself, a compensatory mechanism enabling it to respond to the increasing demands of the pathological features. For instance, decreased activity in the hippocampus is compensated for by the higher activation of other brain areas involved in the response to a cognitive task, such as the frontal lobe. This allows the patient to respond adequately to the task in hand, which does not occur in control groups [59].

Despite advances in the study of neuroplasticity in AD, much remains unknown. Understanding the mechanisms of neuroplasticity degeneration and impairment as well as their behavioral implications and clinical manifestation are key to developing effective pharmacological and non-pharmacological therapies to enhance neuronal plasticity, the strongest correlate of memory and learning impairment in this disease [19,60].

## 3. Current Pharmacological Strategies in AD

Effective therapies for AD, either to prevent or mitigate its symptoms [61], are still notoriously absent or scarce. The pharmacological therapies that are currently available can be categorized according to their main aim, i.e., whether they purport to prevent/delay disease onset and progression or to mitigate symptoms. Disease progression-modifying drugs are only indicated for preclinical or prodromal AD, i.e., stages at which individuals at risk of AD have no or only very slight clinical manifestations of the disease [62]. Drugs that target the clinical stages of the disease, with manifest cognitive symptoms, are indicated for mild to moderate AD (such as donepezil, galantamine, and rivastigmine) or for moderate to severe AD (such as memantine) [63]. There is still insufficient evidence on the efficacy of symptomatic treatment [64]. As therapeutics administered at clinical stages are still scarce and their effects are at most mild, trends are shifting towards targeting the early, prodromic phases of the disease. Further studies are thus urgently required to identify reliable risk factors and AD trajectories in order to develop novel and effective disease-modifying pharmacotherapies [65].

Currently approved drugs for the symptomatic treatment of AD comprise cholinesterase inhibitors and N-methyl-*D*-aspartic acid (NMDA) receptor antagonists [66], both of which provide at best relatively short symptomatic relief; moreover, their efficacy significantly drops as the disease progresses [67]. Of the cholinesterase inhibitors, to date only rivastigmine, tacrine [68], memantine [62], donepezil, and galantamine have been approved for the treatment of AD [67]; aducanumab is the only monoclonal antibody immunotherapy so far (2003) approved by the FDA [68,69,70]. The immunotherapy purportedly hampers Aβ deposition (see recent review by [71]). Clinical trials have shown promising results with other drugs such as Aβ and tau aggregation inhibitors, selective Aβ42 lowering agents, and anti-inflammatory agents, though these trials are still in the initial phases and their safety and effectiveness have not yet been proven [71,72,73,74,75].

Since many of the cognitive dysfunctional signatures of AD involve the cholinergic system, it is not surprising that several of the drugs listed in the preceding paragraph are ligands acting on brain cholinergic circuits. One purported mechanism of action of cholinesterase inhibitors used in AD is the prevention of glutamate neurotoxicity, an effect that is mediated by nicotinic acetylcholine receptors (nAChRs) and the phosphatidylinositol-3-kinase/Akt metabolic cascade [76]. Like other neurodegenerative diseases, AD presents an important chronic neuroinflammatory component [77,78,79]. Methyllycaconitine, an α7-subtype nAChR antagonist, was shown to antagonize the anti-inflammatory effect of nicotine, whereas dihydro-β-erythroidine, an α4β2-subtype nAChR antagonist, had no effect [80]. The homomeric α7 subtype of nAChRs and this metabolic pathway (see review in [81]) are involved in the generation of experimentally-induced neuroinflammation and pro-inflammatory cytokine production [80]. Subsequent work from these authors indicated that increased cholinergic activity in the brain by donepezil prevents experimentally-induced neuroinflammation via the α7-nAChRs/ phosphatidylinositol-3-kinase-Akt pathway, suggesting that this system may form the basis for the development of novel agents for reversing neuroinflammation [82]. As a result of cholinergic dysfunction, cognitive deterioration is also observed in Parkinson disease, another neurodegenerative disorder. The therapeutic strategies aimed at enhancing cholinergic tone in Parkinson disease have been critically reviewed [83].

Galantamine, initially considered an unconventional potentiating ligand of the nAChR [84,85], was subsequently shown to be a low-efficacy agonist acting via a non-orthosteric (agonist) binding site, i.e., an allosteric site on the receptor [86], and to inhibit apoptosis induced by Aβ [87]. Evidence that galantamine augments dopaminergic neurotransmission in the hippocampus through the allosteric potentiation of nAChRs was provided by experiments using a mouse model of Aβ-induced cognitive impairment [88]. These authors postulated that the enhancement of dopamine release may be one of multiple mechanisms underlying the therapeutic benefits of galantamine. Moriguchi and coworkers further showed that galantamine modulated excitatory/inhibitory neurotransmitter equilibrium in the cerebral cortex [89]. Pleomorphic effects of galantamine, combining actions on hippocampal neuroinflammation, deteriorated synaptic performance, and cognitive impairment have more recently been reported [90].

**Figure 2:**
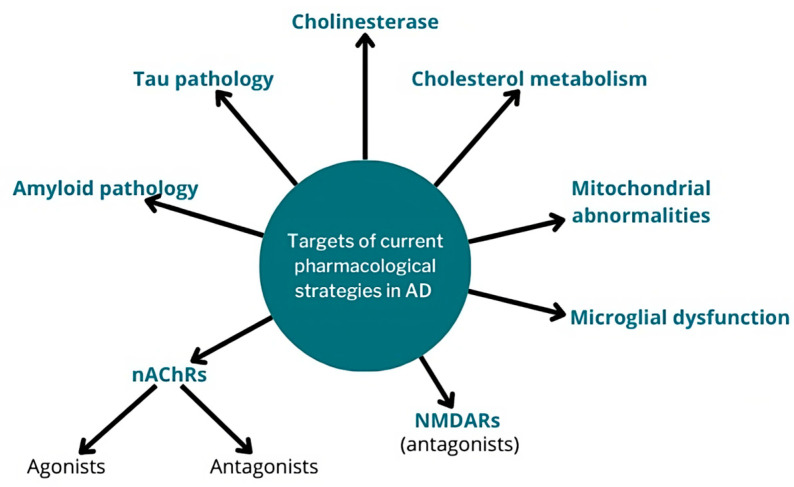
Current pharmacological strategies in AD and their main targets.

Therapeutic strategies that target secondary mechanisms other than amyloid and tau pathologies have also been explored, such as those aimed at mitochondrial abnormalities [91], microglial dysfunction [92], or cholesterol metabolic alterations (such as those employed in the treatment of coronary disease and atherosclerosis), often combined with classical anti-amyloid drugs [93]. A schematic categorization of the current pharmacological strategies is shown in Figure 2.

Some preliminary though promising results have been reported for drugs purported to target neuronal plasticity [94,95]. However, one should keep in mind that such interventions are only effective when there is still an acceptable degree of plasticity in the brain, enabling it to compensate for deficits in functional ability and cognitive status, i.e., when the patients are in the early or even prodromal stages of the disease [96]. There is growing evidence to suggest that interference with adult hippocampal neurogenesis contributes to neurodegeneration in AD [97]; the possibility that AD involves metabolopathies such as dysfunctional brain glucose metabolism [98,99] indicates the use of anti-diabetic drugs as an alternative therapeutic scheme. In AD, glucose uptake was shown to be diminished, thus impairing the brain’s ability to support the required neuronal activity, resulting in cognitive decline. The pro-neurogenic potential of the combined use of the antidiabetic drug metformin and donepezil in a mouse model of neurodegeneration has been reported. Metformin normalized the proteome profile and expression levels of neurogenesis markers along with an improvement in spatial memory. As compared to donepezil, metformin-treated mice exhibited an enhanced number of post-mitotic neurons, suggesting that metformin-mediated adult hippocampal neurogenesis may have implications for the treatment of AD [100]. Other alternative approaches based on plant-derived drugs have been recently reviewed [101]; for instance, the plant extract conophylline was shown to reduce amyloidogenesis and rescue cognitive impairment in a transgenic mouse model of AD [102]. There are also encouraging discoveries of the beneficial effects on cognitive performance of certain compounds found in food. For example, it has been found that in older adults, compounds such as the flavonols found in fruits and vegetables can help protect cognitive function and delay memory impairments in AD [103]. However, it should be noted that most of these advances derive from animal models, and their safety and efficacy remain to be tested in clinical trials.

## 4. Clinical Trials Addressing Neuroplasticity in AD Patients

Most pharmacological agents studied today in the field of AD are aimed at biological processes that promote neuroprotection through a variety of mechanisms [104]. The complex etiopathology and disrupted mechanisms occurring in AD contribute to the failure of recent trials to provide consistent evidence of efficacy, suggesting that a combination of pharmacological approaches rather than monotherapies might perhaps meet with greater success [105].

Clinical trials addressing neuroplasticity in AD are selected based on whether they report measurements related to neuroplasticity, e.g., BDNF levels, synaptic protein levels, changes in functional connectivity measured through MRI, or glutamatergic activity.

Glucose metabolism and insulin are being increasingly researched as possible targets in AD therapeutics. T3D-959 is an anti-diabetic candidate drug that has been recently studied in patients with mild to moderate AD, in a phase 2 clinical trial. T3D-959 is a small-molecule dual agonist of the peroxisome-activated nuclear receptor delta/gamma, also known as PPARδ/γ. The outcome related to neuroplasticity is improved functional connectivity of the hippocampus, as evidenced through fMRI, over the course of three weeks of treatment. Upon correcting insulin resistance in the brain, a change in glucose metabolism was observed, suggesting that insulin signaling, which is commonly affected in AD, is essential for neuroplasticity [106,107]. The administration of insulin itself has also been proposed as a therapeutic agent for AD in a clinical trial that studied its effects on mild AD or amnesic MCI [108]. Although no effects on neuroplasticity were specifically reported by these authors, fMRI measurements showed alterations in cerebral glutamate concentrations upon insulin intake, and glutamate concentrations were hypothesized to be one of the main neuroplasticity-altering mechanisms in AD. A similar study reported changes in glutamate concentration after treatment with empagliflozin, an anti-diabetic that prevents the reabsorption of glucose and favors its excretion [109].

Considering the involvement of glutamatergic neurons and their sensitivity to alterations in AD, it is not surprising many clinical trials have attempted to target the glutamatergic system. The activation of synaptic ionotropic glutamatergic receptors is required for the initiation of plasticity. However, when extrasynaptic glutamatergic receptors are overactivated in AD due to an abnormally high release of glutamate, they produce excitotoxicity and ultimately cell death [110]. Riluzole, currently approved by the FDA for the treatment of amyotrophic lateral sclerosis, is a drug that also targets the glutamatergic system by inhibiting the presynaptic release of glutamate [111]. One phase 2 study addressed the glutamatergic activity through NMR spectroscopy to measure in vivo levels of glutamate and reported a positive correlation between glutamate levels in the posterior cingulate nucleus and cognitive performance, suggesting the potential neuroplastic effect of this intervention in AD [112].

Simulifam, formerly known as PTI-125, is a drug currently studied in phase 3 clinical trials that acts as an Aβ inhibitor and reduces tau hyperphosphorylation. Besides the classical Aβ42 and tau measurements, this study also addresses the CSF levels of neurogranin, a protein present in dendritic spines that is involved in neurogenesis and epigenetic mechanisms of neuroplasticity and is usually considered a biomarker of neurodegeneration [113]. Results of this study showed reduced (32%) levels of neurogranin, suggesting that the protein exerts a potentially protective effect on neurodegeneration [114]. Neflamapinod, an inhibitor of the mitogen-activated protein kinase p38α, was also reported to lower neurogranin levels. However, cognition was found not to be altered in this study, leading the authors to suggest further studies at higher doses [115,116]. The effects of the drug CT1812 have also been studied on other biomarkers of synaptic plasticity such as synaptotagmin and SNAP25 levels, two proteins positively correlated with learning and memory performance [117,118]. CT1812 is an antagonist of the sigma2 receptor, constituting a negative allosteric modulator that could reduce the affinity of Aβ for this receptor, thus inhibiting synaptic toxicity [119,120]. The administration of CT1812 was shown to increase the levels of synaptic proteins and synaptic density [119,121,122].

There are also reports in the literature on drugs that target enzymatic pathways to promote neuroplasticity. Intravenously administered bryostatin, a protein kinase C agonist that is considered a potential therapeutic agent, slightly improved cognitive function in advanced AD patients when compared to placebo [94]. In patients with early-stage AD, 6–12 weeks of oral administration of neflamapinod, a p38α inhibitor, increased episodic memory performance, considered by the authors to be a proxy measure of synaptic function [123]. In patients with mild AD, 24 weeks of treatment with orally administered neflamapimod showed a tendency towards the conservation of episodic memory (but only at high doses), interpreted as an indicator of mildly enhanced plasticity, with a moderate decrease in CSF neurogranin. The authors concluded that longer treatment and higher doses of this drug could be more effective for neuroplastic enhancement [116].

The enzyme glutaminyl cyclase promotes the formation of Aβ oligomers, which exert a toxic effect on synapses, leading to synaptic impairment, reduced connectivity, and a decreased spike number [49,124]. In biomarker-positive AD patients, the administration of PQ912, an inhibitor of this enzyme, reduced neurogranin CSF levels and decreased theta-wave activity in the brain, thus showing the ability of PQ912 to modulate neuronal activity. The authors propose that longer treatment may lead to a more significant disease-modifying effect [125]. Table 2 summarizes the targets.

## 5. Interventions Targeting Neuroplasticity in Animal Models of AD

**Figure 3:**
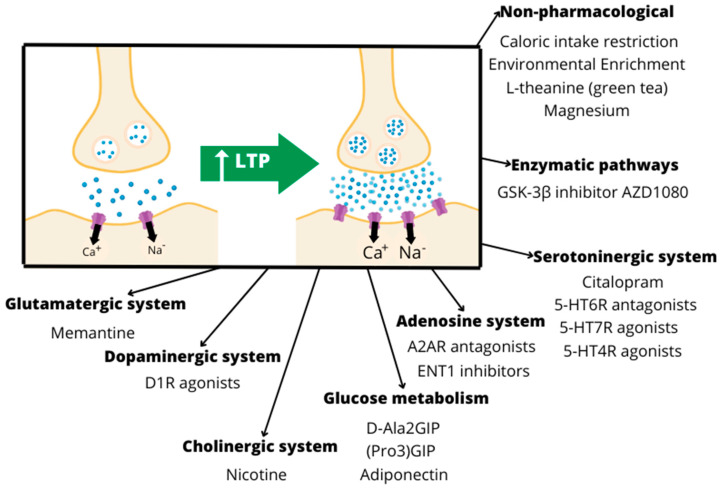
Some of the drugs and mechanisms that enhance LTP and positively affect neuroplasticity.

Animal models provide the opportunity to address neuroplastic mechanisms directly at the level of the cell/tissue, giving them current relevance [126]. Studies reporting pharmacological interventions targeting different mechanisms of neuroplasticity in transgenic or induced AD animal models are categorized according to the main mechanism of action addressing changes in neuroplasticity (Figure 3).

### 5.1. Glucose Metabolism

We have already discussed the relationship between impaired glucose metabolism and insulin deficiency in AD in patients, making these inter-related processes possible therapeutic targets [127]. Furthermore, type 2 diabetes mellitus is a known predisposing or risk factor for AD. Both diseases share the desensitization of brain insulin receptors. The disruption of glucose metabolism and insulin deficiency can lead to neuronal death owing to deficits in energy metabolism, a decrease in neurotrophic factors, and the inhibition of the expression of genes that respond to insulin [128]. The administration of liraglutide, a drug that helps control glucose levels, reversed cognitive impairment in a mouse model and attenuated insulin receptor in a non-human primate model [129]. The administration of metformin was shown to rescue the decreased levels in synaptic protein SYP-1 promoted by the injection of Streptozotocin, used to induce AD in animal models that mimic the sporadic form of the disease [130]. One study analyzed the effect of the anti-diabetic drug sitagliptin in a transgenic mouse model of AD. Sitagliptin increased dendritic spine density, presumably through the BDNF-tyrosine kinase B signaling pathway, as it upregulated the levels of BDNF and tyrosine receptor kinase B (TrkB) [131]. Another study addressed the effects of exenatide, a synthetic analog of the glucagon-like peptide 1 currently employed for treating type 2 diabetes mellitus, on BDNF signaling, and showed a regulatory effect on this pathway [132]. Erythropoietin exerts neuroprotective effects and prevents neurodegeneration and toxicity in nervous cells. Its administration in a mouse model of induced-AD regulates BDNF and PSD-95 expression and attenuates the overexpression of NMDA receptors. Treatment with a NMDA receptor agonist abrogated the positive effects of erythropoietin on neuroplasticity [133].

Glucose-dependent insulinotropic polypeptide (GIP) is a peptide hormone of the incretin family that modulates insulin release and energy utilization, and could be a potential therapeutic factor in AD, in which energy utilization is significantly lower [134]. A novel, long-lasting GIP analog, the glucose-dependent insulinotropic polypeptide analogue (D-Ala2GIP), was found to increase LTP [135], neurogenesis, and synaptic number and plasticity in a transgenic mouse model [136]. GIP and (Pro3)GIP, a similar compound, enhanced LTP and promoted neurogenesis in the hippocampal CA1 region [137].

Lastly, though not an antidiabetic drug per se, tetramethylpyrazine, a compound found in the plant *Ligusticum wallichii* that exhibits powerful anti-diabetic properties, enhanced plasticity in a mouse model of induced AD that simulated the sporadic form of the disease, showing that these effects may not be circumscribed to the genetic, early onset form of AD [138].

### 5.2. Neurotrophic Compounds

Neurotrophins such as nerve growth factor (NGF), glial cell derived-neurotrophic factor (GDNF), and BDNF—the most important neuroplastic-inducing trophic factor—are molecules secreted in the nervous system that are considered to oversee the growth, survival, development, and plasticity of brain cells. It has been shown that BDNF and its receptor, TrkB, are required to consolidate LTP in the dentate gyrus [139]. BDNF increases the trafficking of AMPA receptors in the membrane [140], and is also related to an increase in synaptic density when administered exogenously [141]. The exogenous administration of BDNF has also been related to a novel form of synaptic plasticity in field CA3 of the hippocampus [142].

An alteration in neurotrophic activity is present in the pathogenesis of several neurodegenerative and psychiatric disorders, such as AD, Parkinson disease, Huntington disease, and schizophrenia spectrum disorders [84,142,143]. In AD, a disruption in neurotrophic metabolism leads to impaired neuroplasticity [144,145]. Pharmacological strategies aimed at improving neurotrophic potential have received increased attention of late [145,146]. Several such molecules have been studied in the context of brain aging, such as resveratrol, BDNF, and neurotrophic-type compounds such as rapamycin [146]. BDNF-targeting therapies require further investigation in the context of AD [147]. Studies on the administration of neurotrophic-type compounds include the neurotrophic-derived peptidergic compound (P021) used in a triple transgenic mouse model of AD. The authors reported a marked reduction in the abnormal hyperphosphorylation and accumulation of tau at known major AD neurofibrillary pathology-associated sites. P021 promoted a significant decrease in soluble Aβ levels and a mild tendency towards reduction in Aβ plaque load in the hippocampus, suggesting a reduction in Aβ generation but not its clearance [148]. P021 also had a positive effect on plasticity, but only when administered in the early stages of development (from birth to postnatal day 120): the treatment increased BDNF and ameliorated synaptic protein deficits in a triple transgenic mouse model at up to 4 months of age [149]. It also restored neurogenesis and increased BDNF in the cortex and hippocampus of aged rats, proving to be a potential therapeutic approach in AD as well as in cognitive decline related to aging [150]. Cerebrolysin is a peptide mixture that has neurotrophic effects and has been shown to improve neuroplasticity. Using an amyloid precursor protein transgenic mouse model, cerebrolysin was found to mildly restore neurogenesis by protecting NPC and decreasing the rate of apoptosis [151,152].

A positive allosteric modulator or Trk receptor, ACD856, increased the levels of BDNF in aged mice, adding to the results obtained in vitro demonstrating enhanced nerve growth factor activity and neurite outgrowth and increased levels of the synaptic protein SNAP25 [153].

### 5.3. Glutamatergic System

The activity of neurotransmitters and their receptors is essential to neuroplasticity [154]. To date, the only two families of approved drugs that target neurotransmitters are acetylcholinesterase inhibitors (including donepezil, galantamine, and rivastigmine) and an NMDA antagonist (memantine) [155]. Even though a vast amount of mechanistic knowledge is available on neurotransmitter activity deficits in AD in general, there are still no therapeutic drugs associated with neurotransmitter activity aimed at promoting plastic mechanisms [156].

Aβ exhibits glutamatergic excitotoxic effects: it enhances glutamate release and/or inhibits glutamate uptake by NMDA receptors in neurons and glial cells and increases the influx of Ca^2+^ into the neuron, thus promoting intracellular toxic events. This overstimulation constitutes one of the proposed etiopathogenic mechanisms for AD neurodegeneration [157]. Memantine targets this toxic effect by acting as a moderate affinity open-channel non-competitive inhibitor of NMDA receptors [158], though its efficacy has been questioned as it is not clear whether its therapeutic effects on neurotoxicity can be achieved without affecting cognition. Studies on this topic using animal models report contradicting results [159,160]. These receptors are crucial in LTP mechanisms, raising the question of whether memantine, currently approved for AD treatment, impairs or enhances NMDA receptor-dependent neuroplasticity. Memantine has been reported to rescue LTP impairment induced by soluble Aβ in the dentate gyrus without impairing cognitive performance, though over a certain dose-range it showed disruptive effects on synaptic plasticity and behavior, perhaps because of an excessive blockade of NMDA receptors [161]. Subtype-preferring NMDA receptor antagonists could provide a better and more specific strategy: one study showed that targeting NMDA receptors that contain the GluN2B subunit could prevent the inhibition of plasticity induced by Aβ toxicity [162].

### 5.4. Cholinergic System

Historically, cholinesterase inhibitors, which operate by rapidly degrading the endogenous neurotransmitter acetylcholine, were among the first drugs to be assayed in the context of AD. Today we know that galantamine also acts as a positive allosteric modulator of nAChRs, enhancing neurotransmitter release and Ca^2+^ signaling in neurons [84]. Besides their main effect on the brain, acetylcholinesterase agents can upregulate nAChR biosynthesis in cerebral cortex neurons [163]. This multi-target pharmacological effect is also shared by donepezil-related compounds [164]. The cholinergic hypothesis is a theoretical construct that provided the basis for employing anticholinesterase drugs in AD [165,166], the efficacy of which is still under debate [167]. Despite Tacrine being withdrawn from the market owing to its hepatotoxicity, the drug appears to improve cognitive performance in an AD transgenic animal model. Tacrine also increased the levels of NMDA receptor subunits NMDAR2A, NMDAR2B, and the synaptic-associated proteins PSD-95 and SYP [168]. Donepezil together with cerebrolysin showed a synergistic and protective effect on plasticity, promoting a wider dendritic arborization in pyramidal neurons of the prefrontal cortex, dorsal hippocampus, nucleus accumbens, and dentate gyrus [169].

**Figure 4:**
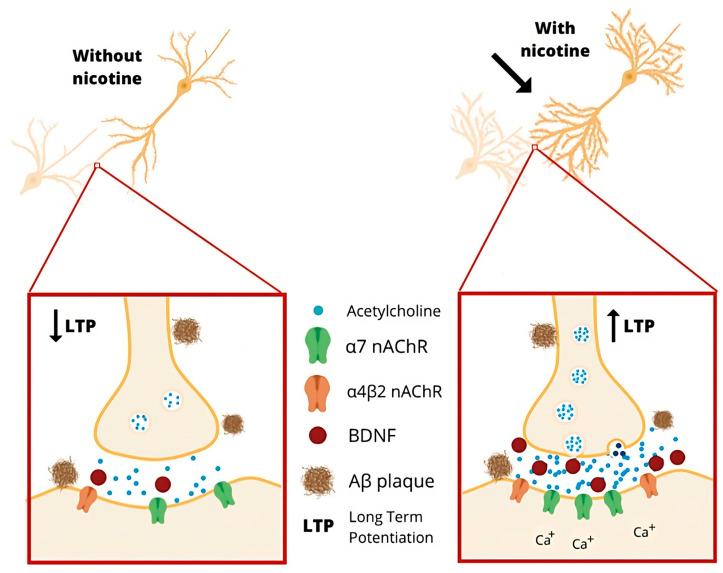
The chronic administration of nicotine is purported to increase LTP via cholinergic receptors [167].

Chronic nicotine administration has been shown to prevent Aβ-induced inhibition of synaptic transmission and LTP in the hippocampus; to downregulate α7 and α4 nAChRs, presumably by increasing BDNF levels [170]; and to increase dendritic density in the CA1 area of the hippocampus when administered chronically [171]. α7 nAChRs are considered a potential target owing to their essential role in different mechanisms of synaptic plasticity [172,173] (Figure 4).

### 5.5. Serotoninergic System

The serotonin 5-HT4 receptor participates in memory and learning processes and mechanisms of plasticity such as LTP. One study reported that in transgenic rats, the administration of BIMU8, an agonist of this receptor, not only improved cognitive deficits but also increased LTP in the hippocampus [174]. Fluoxetine is an antidepressant drug currently available on the market that has been found to exert neuroprotective and neuroplastic effects [175], but its effects on AD have not been sufficiently studied to date. In transgenic AD mice, the administration of Fluoxetine in early stages (adolescence) attenuates cognitive and synaptic deficits in the adult animals [176].

Citalopram is another antidepressant that can ameliorate Aβ production and deposition in AD mice and human brains. Additionally, the administration of citalopram in an animal AD model was shown to reverse Aβ-induced LTP impairment in the hippocampus. Its effect on LTP was explained by two mechanisms: (1) a restoration of the number of 5-HT receptors, which increases serotonin levels and restores LTP, and (2) a decrease in the levels of Aβ accumulation in the hippocampus, which is known to inhibit LTP [177]. LTP was also rescued by the chronic administration of the serotonin type 6 receptor (5-HT6R) antagonist [178] and by the chronic administration of a serotonin type 7 receptor (5-HT7R) agonist [179], in a rat model.

### 5.6. Dopaminergic System

The dopaminergic system is also involved in the regulation of plasticity, though its role in AD is still not clear. It has been reported that the malfunction of dopaminergic activity induces LTD and suppresses LTP, generating memory impairment [180]. Studies on therapeutic pharmacological strategies addressing the dopaminergic system in AD are scarce; it has been reported that an agonist of the D1-type dopamine receptor that also acts as a D2-type receptor antagonist improved hippocampal-dependent learning and memory and increased LTP in the hippocampus by improving the surface expression of GluA1-containing AMPA receptors [181]. Other studies have addressed the dopaminergic system in aged animals, but not specifically in AD [182].

### 5.7. Adenosine System

Adenosine is a homeostatic modulator of various physiological processes, including sleep and cardiac and cognitive functions [183]. Adenosine interacts with G-protein coupled receptors throughout the brain, thus contributing to neuronal signaling and cognition. Dysregulation of A_2A_ adenosine receptors is observed in some AD patients. When compared to healthy controls, these receptors are significantly upregulated in the hippocampus and cortex, impairing the regulation of pro-inflammatory cytokine secretion associated with neuroinflammation [184,185]. The activation of hippocampal A_2A_ regulates plasticity, especially glutamate release and NMDA receptor activation [186]. A_2A_ antagonists have been studied in animal models and are reported to normalize the upregulation of A_2A_ receptors, increase the expression of the synaptic markers syntaxin-1 and vGluT1, restore LTP amplitude, and improve cognitive performance [187]. Blocking these receptors inhibited the facilitation of LTP in hippocampus through BDNF [188].

Adenosine levels can also be increased by inhibiting the adenosine equilibrative nucleoside transporter 1 (ENT1), which oversees adenosine recycling from the extracellular space. In a transgenic mouse model of AD, an inhibitor of ENT1 was able to restore LTP and the levels of the glutamate receptor subunits NR2A and NR2B [189].

### 5.8. Enzymatic Pathways

In the brain, the enzyme protein kinase C (PKC) participates in the regulation of neurotransmitter release, cell proliferation and differentiation, gene expression, and neuroplasticity. PKC is involved in the development of AD pathophysiology through the alteration of its signaling pathways, which are associated with a decline in episodic memory [190]. Bryostatin, an activator of the PKC epsilon (PKCε) isozyme, has been demonstrated to restore synaptic and neuronal loss in transgenic mice at a stage akin to pre-clinical AD [191].

The presence of the α isoform of the p38 mitogen-activated-protein kinase (p38α) in neurons promotes inflammation, Aβ formation, and synaptic dysfunction, thus mediating age-related cognitive decline [192,193]. The inhibition of this protein serves to protect synapses and cognition in transgenic animal models of AD, thus constituting an additional target for synaptic pathology in this disease [194,195,196,197]. Another enzyme that has been targeted for AD treatment is glycogen synthase kinase-3β (GSK-3β). Inhibiting this enzyme could lead to the prevention of tau phosphorylation, a typical occurrence in postmortem AD brains. AZD1080 has been proposed as a selective GSK-3β inhibitor, which has been studied both in vitro and in vivo. AZD1080 inhibited tau phosphorylation in fibroblasts in culture, while in mice it reversed memory impairment and prevented LTP disruption when administered sub-chronically, but not acutely [198].

Berberine, the main active component of several herbs used in traditional Chinese medicine, has recently been proposed as a therapeutic strategy in AD. Berberine regulates the GSK-3β/PGC-1α signaling pathway by inhibiting GSK-3β activity [199], showing potential neuroprotective effects against oxidation, neuroinflammation [200], Aβ pathology, and tau hyperphosphorylation [200,201]. It has also been suggested that berberine modulates the extracellular signal-regulated kinase and protein kinase B signaling pathways in a transgenic model of AD, thus regulating plasticity, as the activation of this signaling pathway is related to the mechanism of neuroplasticity [202]. Another signaling pathway that is altered in AD is phosphoinositide dependent kinase 1 (PDK1)/AKT, which is involved in AB production and tau phosphorylation, as well as in cell survival and synaptic health [199]. Therapeutic strategies attempting to activate this signaling pathway include Salvia officinalis [203], curcumin [204], and trypchloride [205]. All these compounds have been reported to produce a certain degree of cognitive enhancement in animal models of AD and reduce neuropathology [202,206]. However, safety factors and bioavailability need to be further investigated [207].

### 5.9. Neuroinflammation

Metabolic pathologies constitute risk factors for AD, and it has been proposed that metabolic dysregulation, like insulin resistance, is a precursor to AD (especially the sporadic form, late onset AD). Several metabolopathies are comorbidities of AD. Metabolic diseases are intimately linked to the production of inflammatory cytokines and the accumulation of AD pathological byproducts in the brain, which is why compounds with anti-inflammatory action are being currently considered as possible therapeutic strategies [208]. Chronic metabolic stress and dysregulated AMP-activated protein kinase have been associated with the development of neurological diseases and aberrant neurogenesis [209,210]. A proinflammatory cytokine that has been targeted in AD is tumor necrosis factor-α (TNF-α), which is typically elevated in patients and animal models of AD. One such drug, 3,6’-dithiothalidomide, was studied in a transgenic AD model. It was demonstrated that this compound was able to increase the levels of synaptic protein SNAP25 and synaptophysin, which indicates a preserved synaptic function, and to enhance cognitive impairment [211].

Interleukin-1β is another pro-inflammatory cytokine whose expression is higher in AD patients. An inhibitor of the nucleotide-binding oligomerization domain-like receptor family, pyrin domain containing 3, dapansutrile, was shown to rescue LTP, though only at high doses [212]. Another cytokine that regulates inflammation, interleukin-2 (IL-2), was found to increase synaptic density in a transgenic mouse model of AD [213].

Sodium butyrate is an inhibitor of histone deacetylase and reduces the secretion of pro-inflammatory cytokines. A study investigating its effect after two weeks of administration reported improved plasticity as shown by increased LTP, higher dendritic density, and preserved levels of synaptic-related proteins PSD-95, SYP, and NR2B [214].

### 5.10. Lipid Metabolism

The strongest genetic risk factor for AD is the presence of the apolipoprotein E (ApoE) allele epsilon 4 (*APOE4*), which is present in roughly 50% of all cases, albeit with important ethnic variability [215]. ApoE4 is involved in lipid metabolism, most importantly in cholesterol transport, and cholesterol dyslipidemias are thought to be involved in AD pathogenesis [216]. It is believed that ApoE has a crucial role in generating Aβ protein, which in turn leads to defective neuronal sprouting and dysfunctional plasticity, synaptic loss, and ultimately, neurodegeneration [217,218]. ApoE4 impairs the function of NMDA glutamatergic receptors, and is involved in the metabolism, aggregation, and toxicity of Aβ peptide, tauopathy, synaptic plasticity, lipid transport, glucose metabolism, mitochondrial function, vascular integrity, and neuroinflammation, although the underlying processes are not well understood [219]. Therapeutic drugs that focus on modulating ApoE activity include statins, estrogen, anti-inflammatory drugs, and antioxidants [220,221]. Probucol, a cholesterol-lowering drug which induces ApoE production and peripheral circulation of this lipoprotein and one of its main receptors, LRP, increases the synthesis of the rate-limiting enzyme in cholesterol synthesis, reduces age-related glial activation, and induces the production of the synaptic marker SNAP-25, suggesting a potential role in supporting plasticity [222]. Another therapeutic strategy is to activate phospholipases, a group of enzymes that hydrolyze phospholipid substrates and protect from synaptic dysfunction and cognitive deficits [223,224,225]. Gene therapy has been proposed as a strategy to regulate cholesterol homeostasis by targeting 24-dehydrocholesterol reductase, which is usually downregulated in AD. This approach was tested in animal models. No outcomes related to neuroplasticity have been reported as yet [226].

Cholesterol is crucial for the regulation of nicotinic receptors in neuronal membranes, especially the α7 and α4 nAChR subtypes, which, as mentioned previously, are fundamental regulators of neuroplasticity and cognitive function, and their expression at the surface is essential for the correct functioning of the cholinergic neuron [216]. In cultured rat neurons, the statin-lowering drug lovastatin showed a regulatory function by modulating protein receptor levels at the cell surface [227]. Statins have pleiotropic actions, including immunomodulatory, anti-inflammatory and antioxidant effects that could also protect neurons in AD [228,229].

Adiponectin is an adipokine that regulates lipid metabolism, among other functions; it has recently been proposed that changes in its expression could be related to an increased risk of developing AD [230,231]. Adiponectin has been tested in a transgenic model of AD and been shown to rescue LTP [232,233].

## 6. Conclusions

Therapeutic strategies for AD are highly diverse, as are their putative mechanisms of action and the presumed etiopathogenic mechanisms they address. Here we have summarized the pharmacological strategies aimed at improving neuroplasticity and their reported outcomes in both clinical settings and animal model studies. The enhancement of LTP reported in several of these studies provides a positive scenario for future research addressing the correlation between neuroplasticity and improvements in the cognitive performance of AD patients. Whilst several studies in animal models show promising results, clinical trials are more discouraging, many reaching the conclusion that the efficacy of the therapeutic treatments is at best mild to moderate and suggesting that longer treatment or higher doses would be required to achieve significant changes in neuroplastic markers. Intervention in the early stages of the disease would therefore appear to be determinant, thus taking advantage of the fact that the brain still retains some plasticity despite the initial development of pathological hallmarks. It should also be taken into account that most of the experimental models providing encouraging results are carried out using transgenic animal models of AD, which are closer to the model of familial AD, whereas clinical trials mostly consider cases of sporadic AD, without the genetic alterations addressed in transgenic models. This could explain the differences between the two scenarios and should be addressed in future studies.

## Figures and Tables

**Table 1:** CSF Markers of Altered Neuroplasticity in AD.

Protein	Process in Which It Is Involved	Mode of Presentation in AD
Neurogranin	Promotion of synaptic plasticity and LTP	Increased
SNAP-25	Control of synaptic plasticity	Increased
BDNF	Structural plasticity and cognitive plasticity/learning	Decreased
VEGF	Neurogenesis and synaptic plasticity	Decreased

**Table 2:** Examples of neuroplastic targets, drugs, and expected outcomes in AD therapeutics.

Target	Drug	Outcome
Glucose metabolism	T3D-959 (anti-diabetic)	Improved functional connectivity of the hippocampus
Glucose metabolism	empagliflozin	Alleged neuroplasticity improvement through glutamate activity regulation
Glutamatergic toxicity	Riluzole	Inhibition of the presynaptic release of glutamate and associated glutamatergic toxicity
Amyloid and tau pathologies	Simulifam	Decrease in synaptic neurogranin levels
Mitogen-activated protein kinase p38α	Neflamapinod	Decrease in neurogranin levels/ Enhanced memory performance
Sigma2 receptor	CT1812	Prevention of synaptotoxicity induced by Aβ, increased levels of synaptic proteins related to plasticity, and synaptic density
Protein kinase C	Bryostatin	Enhanced cognitive performance
Glutaminyl cyclase	PQ912	Decrease in neurogranin levels
